# The comparison of the antioxidant, antibacterial and antiviral potential of Polish fir honeydew and Manuka honeys

**DOI:** 10.1038/s41598-024-82429-0

**Published:** 2024-12-28

**Authors:** Dorota Grabek-Lejko, Michał Miłek, Małgorzata Dżugan

**Affiliations:** 1 Department of Bioenergetics, Food Analysis and Microbiology, Institute of Food Technology and Nutrition, University of Rzeszow, Zelwerowicza 4 Street, Rzeszow, 35-601 Poland; 2 Department of Chemistry and Food Toxicology, Institute of Food Technology and Nutrition, University of Rzeszow, Ćwiklińskiej 1a St, Rzeszow, 35-601 Poland

**Keywords:** Honeydew honey, Manuka Honey, Antiviral, Antibacterial, Bacteriophages, Antioxidants, Polyphenols, GOX, Hydrogen peroxide, Microbiology, Health care

## Abstract

The aim of the present study was to compare the antioxidant, antibacterial and antiviral activities of Podkarpackie coniferous honeydew honey and Manuka honey. The quality of tested honey samples (honeydew-12 and Manuka-4) regarding honey standard was evaluated as well as additional indicators (methylglyoxal, total phenolics and HPTLC phenolic profile, antioxidant potential, glucose oxidase activity, and hydrogen peroxide) were compared. Antibacterial potential was analyzed against Gram-positive (*S. aureus* and *B. cereus*) and Gram-negative (*E. coli* and *S. enterica*) bacteria. Antiviral activity against different RNA (phi6, MS2) and DNA (T7, phiX174) bacteriophages considered as “viral surrogates” was determined. Based on the determined physicochemical parameters the good quality of tested honeys was confirmed, excluding two samples. The content of polyphenolic compounds in honeydew honey ranged from 583.87 to 1102.42 mg of gallic acid/kg and was strongly correlated with the antioxidant properties. Moreover, for samples with the strongest activity these parameters were comparable to Manuka honey. However, the obtained HPTLC polyphenolic profiles were completely different for honeydew than for Manuka honey which exhibited additional bands (Rf = 0.74 and 0.52). Honeydew honeys were characterized by a strong antiviral and antibacterial properties most of all against Gram-positive bacteria. The MICs (minimal inhibitory concentrations) for *S. aureus* and *B. cereus* ranged 15–35% and 8–15% for honeydew and Manuka honeys, respectively. The strongest antiviral properties of honeydew honey were demonstrated mainly against RNA bacteriophages (phi6, MS2) which was even higher than for Manuka honey, especially against MS2 virus. The obtained results suggest that Podkarpackie honeydew honey with the controlled glucose oxidase activity may be a natural substance used to combat viral and bacterial diseases.

## Introduction

Bacterial and viral infections are very common in the world and 7.7 million people die from bacterial infection each year^1^. Because viral diseases are hard to quantify they take a significant toll of human life^2^ and viral infections pose unquestionably the greatest threat of a pandemic in the modern period^3^. There are many antibacterial and antiviral drugs on the market. However, due to their cytotoxicity, and the development of pathogen resistance, difficulties in treating drugs arise. Considering the limitations of conventional drugs, it has become critical for researchers to investigate natural substances with antimicrobial and antiviral effects^4^.

Honey is one of the oldest natural products which has been used in traditional medicine for centuries (around 5 500 years) not only for its nutritional purposes but also in the prevention and treatment of many diseases. The scientific evidence of the antibacterial activity of honey was first reported by Van Ketel in 1892^5^. Nowadays, *in vitro* studies show that honey is widely recognized as a suitable therapeutic agent for its antimicrobial, anti-inflammatory, antioxidant and anti-tumor properties. However, its biological potential and mechanisms of action has not been understood completely, and are still under examination and requires confirmation in clinical trials in the future^6^. It is known that the content of active substances and honey bioactivity varies and its potential depends on the biological activities of the initially harvested floral source, its geographical origin, variety, season, storage conditions, health of bee colonies and suitable beekeeping practices^7,8^. In general, dark honeys possess higher biological properties than light honeys and honeydew honey can be mentioned as one of the most valuable dark honey varieties. This honey is produced by bees (*Apis mellifera*) from secretions of living parts of plants or excretions of plant-sucking insects and possesses different characteristics than blossom honey which is produced from the flower nectar. Honeydew honey is mainly produced from coniferous trees, such as fir (*Abies alba*), spruce (*Picea abies*) and can be also produced from leafy trees such as oak and lime^9^. Podkarpackie, Southeastern region of Poland has long traditions in beekeeping, the first sources come from 15-th century. This ecologically clean region is rich in coniferous forests dominated by fir trees and it is an excellent place where honeydew honey can be collected. Since 2010 Podkarpackie honeydew honey has been registered by the European Commission as a protected destination of origin (EU quality label) (https://ec.europa.eu/agriculture/eambrosia/geographical-indications-register/)^10^. The dark color of Podkarpackie honeydew honey comes from the type of fir present in this area, and this honey possesses higher than normative content of simple sugars and moderate acidity, which influences on the taste bouquet of honey. Honeydew honey is usually high in bioactive compounds such as minerals (Mg, Mn, Fe, Co, Cu, Ca, P), phenolics, proteins, amino acids, and enzymes and also has higher antioxidant and antimicrobial activities than blossom honeys^11,12^.

In turn, the world-famous Manuka monofloral honey, originating from New Zealand and Australia, is made from the nectar of Manuka myrtle (*Leptospermum scoparium*). It is unique, because of its high methylglyoxal (MGO) content, which is responsible for its antibacterial potential^13^. Manuka honey is one of the few types of honey approved for medicinal use to treat infections^14^. However, while the Manuka honey is considered as the most active honey, recent studies on the antibacterial properties of different honey types produced over the world have reported similar or even superior antibacterial efficacy compared to Manuka honey^15^.

Searching for natural products with possible antiviral potential becomes very important, because of our experience in the last pandemic and the increasing interest of people in natural products treatment^4^. Since some viruses are technically challenging to study *in vitro*, bacteriophages can be used as substitutes, to mimic pathogenic viruses. Among them, a double stranded RNA (dsRNA) enveloped phi6 bacteriophage non-pathogenic to humans has been well-studied as safe surrogate for pathogenic enveloped viruses such as SARS-CoV-2 virus (structurally similar), influenza virus and Ebola virus. Lipid-based envelope of this virus is to provide resistance to antiseptic and protect the viral envelope^16^. Phi6 is a biosafety level 1 (BSL-1) plant pathogen, culturable in laboratory conditions without posing any human health risk. Similarly, MS2 is a well-known bacteriophage with a structure and function similar to many human enteroviruses (polioviruses 1–3, coxsackieviruses, echoviruses and enteroviruses 68–72) and can be used as a surrogate for small human infective RNA viruses (e.g., Ebola virus, Marburg virus, and equine encephalitis alphaviruses)^17,18^. Moreover, enteric viruses are a major problem in the food industry, especially human noroviruses are the leading cause of nonbacterial gastroenteritis^19^. In turn, bacteriophages phiX174 (ssDNA) and T7 (dsDNA) are used as a model of DNA viruses. They are considered as suitable viral surrogates for studying viruses that infect eukaryotic cells as they present similar structural characteristics and are safe to use^20–22^. Moreover, bacteriophages are relatively easy to produce in large quantities and suitable for antiviral studies^22^. Taking into account, the main objective of this study was to compare the *in vitro* antioxidant, antibacterial and antiviral activity of Podkarpackie honeydew and Manuka honeys. Moreover, an extensive set of honey parameters was tested to find those responsible for honeydew honey bioactivity. According to our knowledge, no studies have been conducted on the antiviral effects against used set of bacteriophages of Polish honeydew honey. Hence, the outcomes of this study could provide useful information on the potential of Podkarpackie honeydew honey as an antiviral and antibacterial agent for the health sector.

## Materials and methods

### Material

Twelve honeydew honey samples were collected from the apiaries located in the Podkarpacie region (the Southeastern part of Poland) in 2021 and 2022. Four Manuka honeys, with the different MGO (methylglyoxal) content (100, 250, 400 and > 550 mg/kg) were purchased from the local food shop. The melissopalynology was used to confirm the botanical origin of Manuka honey. The samples were stored in the dark at room temperature (22 ^o^C±2.0) until analysis (up to 6 months).

### Physicochemical parameters of honey samples

The pH value of 20% aqueous honey solutions were measured using SevenCompact™ S210 pH-meter (Mettler Toledo, Columbus, OH, USA) at a temperature of 25 ^o^C±2. The free acidity was determined using the standard alkali titration method (0.1 M NaOH) up to pH = 8.3, controlled using pH-meter. The results were presented as mval/kg of honey.

Conductivity was measured in 20% aqueous honey solutions using a conductometer CP-401 (Elmetron, Zabrze, Poland). The results were expressed in mS/cm.

The water content was determined by the refractometric method, using a digital refractometer (HI96800; Hanna Instruments, Woonsocket, RI, USA).

The honey color was measured colorimetrically using the HI 96785 colorimeter (Hanna Instruments, Woonsocket, RI, USA) and the results were expressed in the mm of Pfund scale.

The HMF content (HMF) was determined in 20% aqueous solutions using the reflectometric method (RQflex20, Merck, Darmstadt, Germany) according to the manufacturer’s instructions.

Diastase number (DN) was determined by a spectrophotometric method using the Phadebas Honey Diastase Test (Magle AB, Lund, Sweden) according to the manufacturer’s instructions, written in detail by Dżugan et al.^23^.

### Ferric reducing antioxidant power (FRAP)

The antioxidant potential was determined according to Benzie et al.^24^ method and adjusted to 96-well plates^25^. Trolox (Sigma Aldrich Co., USA) was used for the calibration curve and the results were expressed as µmol of Trolox per kg of honey.

### Total phenolic content

The content of phenolic compounds was determined with a spectrophotometric method using the Folin–Ciocâlteu reagent (Sigma-Aldrich, Saint Louis, MO, USA) described in detail by Grabek-Lejko and Hyrchel^25^. The results were expressed in mg of gallic acid equivalent (GAE) per kg of honey.

### HPTLC polyphenolic profile analysis

The analysis of phenolic profiles has been performed for methanolic extracts of honeydew and Manuka honey samples obtained by solid phase extraction (SPE). Twenty grams of honey was dissolved in acidified (pH 2.0) deionized water and passed through C-18 Sep Pack Cartridge (Waters Co, Milford, MA, USA) columns previously conditioned with methanol and acidified water. The sugars were then removed by rinsing with pH 2.0 water and the methanol extracts were collected. Chromatographic analysis was performed using the Camag HPTLC kit (Camag, Muttenz, Switzerland) as described by Tomczyk et al.^11^. The eluent chloroform: ethyl acetate: formic acid (50:40:10 v/v/v/v) and p-anisaldehyde/sulfuric acid reagent was used to develop the plate.

### Glucose oxidase activity (GOX)

GOX was determined with a Megazyme GOX assay kit (Megazyme International Ireland Ltd, Bray, Ireland). This method is based on the oxidative catalysis of glucose to D-glucono-δ-lactone and hydrogen peroxide. In the next step, H_2_O_2_, in the presence of peroxidase, reacts with p-hydroxybenzoic acid and 4-aminoantipyrine to form a quinoneimine dye complex, measured spectrophotometrically at 510 nm. For this purpose, fresh 20% (w/v) honey solutions were used and GOX activity was determined in a 96-well microplate, according to the manufacturer’s instructions and expressed as mU of GOX/ml of honey.

### Hydrogen peroxide content

Hydrogen peroxide (H_2_O_2_) was determined by using horseradish peroxidase (HRP) –o-dianisidine colorimetric method according to Lehman et al.^26^ based on the reaction of hydrogen peroxide with o -dianisidine in the presence of horseradish peroxidase type II to form a colored product (brown color). Oxidized o-dianisidine reacts with sulfuric acid to form a more stable colored product (pink color). The intensity of the pink color measured at 560 nm is proportional to the original hydrogen peroxide concentration. The hydrogen peroxide content was determined after 2 h, 4 h, 6 h and 24 h of incubation at 35 ^o^C in an orbital shaking incubator at 180 r.p.m. For the calibration curve, different concentrations of hydrogen peroxide (Sigma Aldrich Co., USA) were prepared and results were expressed as µM of H_2_O_2_ in a 25% honey sample.

### Antibacterial properties—broth microdilution method, MIC, MBC

Antibacterial potential of honey was measured by using the broth microdilution method described by Grabek-Lejko et al.^27^. Antibacterial properties of honey were determined against Gram-positive bacteria: *Staphylococcus aureus* ATCC 25923, *Bacillus cereus* PCM 482, and against Gram-negative ones: *Escherichia coli* NCTC 1893 and *Salmonella enterica* subsp. *enterica* serotype Enteritidis NCTC 12634. For this purpose, overnight bacterial cultures grown on TSA (Trypticaseine Soy Agar, Biomaxima, Poland) were suspended in phosphate-buffered saline (PBS), pH 7.2 to the final optical density 0.132 (measured at 600 nm, UV-VIS spectrophotometer Hach DR 6000, (Hach Co., Loveland, Co, USA) which corresponds to the 0.5 McFarland turbidity scale (around 1-1.5 × 10^8^ CFU/ml). Then bacterial suspensions were diluted 150x times with a double concentrated Mueller-Hinton Broth medium (MHB, Biomaxima, Lublin, Poland) to the final concentration of bacteria around 1 × 10^6^ CFU/ml. Then, 100 µl of bacterial suspension was mixed with 100 µl of different concentrations of honey samples (4,6,8,10,15,20,25,30,35,40,45,50% w/v) in 100-well honeycomb microplates and incubated with shaking at 37^o^C for 24 h in a Bioscreen C apparatus (Oy Growth Curves AB, Ltd., Helsinki, Finland). As a positive control MBH with bacteria and without honey addition was prepared. As a negative control MBH medium (without bacteria and honey) was used. The optical density of bacterial growth was determined every 1 h. The results were expressed as bacterial growth curves for 15, 20 and 25% of honey samples and as MIC and MBC values. The MIC 90 value (minimal inhibitory concentration of honey which inhibits bacterial growth in ≥ 90%) was determined in comparison to the corresponding positive control after 24 h of incubation. MBC (Minimum Bactericidal Concentration) was determined by pouring 5 µl from each well of the broth microdilution method on the TSA medium and incubation of the plates at 37 ^o^C for 24 h. The MBC value was recorded as the lowest concentration of honey at which no survival of viable bacteria was observed.

### Antiviral potential of honey

Antiviral potential of honey was analyzed against four bacteriophages (viral surrogates) according to Grabek-Lejko et al.^28^. The bacteriophages used for research differed in genetic material, structure (having a capsule or not) and their characteristics are presented in Table 1. All bacteriophages and their bacterial hosts were purchased from Leibniz-Institute DSMZ, Deutsche SammLung von Mikroorganismen und Zellkulturen GmbH (Braunschweig, Germany). The replication of bacteriophages was performed according to Grabek-Lejko et al.^26,27^. The double agar overlay plaque method was used for the determination of antiviral potential of honey. For this purpose, 10 µL of analyzed phage was mixed with 100 µL of different honey solutions and incubated at room temperature at different times (2,4,8,12,24 h). As positive controls, phages in STM buffer (50 mM Tris-HCl pH 7.5, 0.1 M NaCl, 8.1 mM MgSO_4_, 0.01% (w/v) gelatin) were used. After incubation, 0.010 mL of serially diluted samples were mixed with 100 µL of overnight culture of bacterial hosts. For this, *P. syringae* was incubated on TSA medium, at 25 °C and the bacterial concentration used was 0.25 (OD 600 nm), while *E. coli* hosts were incubated on LBA medium at 37 ^o^C and the final bacterial concentrations used were 0.115 (OD 600 nm). Then, samples were mixed with 5 mL of semi-solid appropriate medium (TSB or LB) and poured onto the TSA or LBA plates (Table 1). After 24 h of plates incubation, the number of viable bacteriophage particles (plaques) were counted and the results were expressed as log_10_ PFU (plaque-forming units) per mL of the sample. The antiviral potential of honey was analyzed by comparing the quantity of PFU with the control samples (pure bacteriophages in STM buffer, without honey addition).

**Table 1:** Surrogate viruses used in this study^29^.

Bacteriophage	Phi6 DSM 21518	MS2 DSM 13767	T7 DSM 4623	PhiX174 DSM 4497
Host	*P. syringae* DSM 21482	*E. coli* DSM 5695	*E. coli* DSM 613	*E. coli* DSM 13127
Genome	dsRNA	ssRNA	dsDNA	ssDNA
Family	*Cystoviridae*	*Fiersviridae*	*Autographiviridae*	*Microviridae*
Morphology capsid	Enveloped spherical virion covered by spikes hexagonal icosahedral	Non-enveloped, isometric, icosahedral	Enveloped, icosahedral	Non-enveloped, icosahedral
Size (nm)	60–100	25	60-head, 19-tail	25
Growth conditions	25 °C, TSA, semi-solid TSB + 0.7% agar	37 °C, LBA, semi-solid LB + 0.7% agar	37 °C, LBA, semi-solid LB + 0.7% agar	37 °C, LBA, semi-solid LB + 0.7% agar

*TSA* Trypticasein Soy Agar, *TSB* Trypticasein Soy Broth, *LBA* Luria Bertani Agar, *LB* Luria Bertani.

### Statistical analysis

All measurements were carried out in triplicate. The significance of differences was tested after ANOVA analysis using Tukey’s post hoc test (*p* = 0.05). All calculations were made using Statistica 13.3 software (StatSoft, Tulsa, OK, USA). The differences in the performance of honeydew honey samples compared to Manuka honey were assessed using the t-test.

## Results and discussion

### The quality of tested honeydew and Manuka honeys regarding legal standard

The basic physicochemical parameters of honey are summarized in Table 2. Obtained values were verified regarding the requirements of the quality of honey included in the European Union directive from 2014 (Directive 2014/63/EU)^30^. The tested honeydew honeys met the regulatory requirements well in terms of all determined parameters, excluding conductivity and diastase number. According to this directive, the honeydew honey can be classified by the electrical conductivity, with the value higher than 0.8 mS/cm. In the most analyzed samples (83% of total) the conductivity value was above 0.8, with the exception for samples no 1 and 2 which also contained elevated HMF and lower diastase number (DN).

HMF (hydroxymethylfurfural content) shouldn’t exceed 40 mg/kg and higher values of HMF may be related to a long, inappropriate honey storage, long heating or adulteration with corn syrup^31^.

According to the regulatory standards, the DN number must be equal or higher than 8, with the exception for honeys with naturally low enzyme content, for which the DN number must be not lower than 3^32^. Among analyzed samples, only the sample no 2 (DN 4.62) did not meet the requirements. The diastase number of honeydew honey samples ranged from 12.4 to 23.48 and was similar as previously described 12.5–17.45^33^ and 10.04–14.64^9^. This can suggest that samples 1 and 2 may have been overheated. However, based on the beekeeper declaration we did not exclude these samples from the further study but we considered them as a negative control. The study confirmed, that biological properties (antibacterial, antiviral and antioxidant) of these samples were much weaker, which further emphasizes the importance of performing basic physicochemical analyzes and proper classification of honey.

Comparison of parameters for honeydew and Manuka honeys indicates that, despite the observed high variability within the groups, significant differences between the both groups occurred for diastase number, color, acidity and conductivity. The DN values of honeydew honeys were much higher than Manuka samples, which proves the more beneficial enzymatic activity of Polish honeys. However, the low DN for Manuka honey was also observed by other authors^34,35^. It was observed that MGO and 3-phenyllactic acid, present in Manuka honey, accelerate loss of diastase and this parameter is not a good indicator of heat treatment or inappropriate storage of Manuka honey^34^. It is known that honey with naturally low enzymatic activity is characterized by a DN value between 3 and 8 Schade units and an HMF below 15 mg/kg^34–37^, which is also observed in tested Manuka honey samples. Based on these guidelines, all samples met the requirements of the standard.

The color of honey depends on its chemical composition, like content of phenolics, carotenoids, minerals, pollens, floral and geographical origin and also time and temperature of honey handling, storage, processing^38^. Based on a Pfund scale of honey color, USDA (United States Standards for Grades of Extracted Honey. USDA, Agricultural Marketing Service. Effective May 23, 1985) classified honey into seven categories of color: water white, extra white, white, extra light amber, light amber, amber and dark amber^39^. According to the obtained results, the analyzed honeydew honey samples can be classified as amber (with the values from 86 to 114 mm Pfund scale) to dark amber (with the values > 114 mm Pfund scale). Similar classification was reported by Bodor et al.^38^ who examined honeydew honey from Hungary and observed that color values were within the range of 58 to 194 mm Pfund scale. Color of pine honeydew honey from Greece ranged from 60 to 118 mm Pfund scale, which can be classified as light amber, amber and dark amber^40^. Compared to honeydew honeys Manuka honey samples were characterized by darker and less differentiated color. All samples can be classified as dark amber.

The pH and acidity and water content of Manuka honeys were lower than those of honeydew. However, Tomczyk et al.^11^ reported also the high acidity of honeydew honeys in the range 37.70–65.50 mval/kg, with the average value of 48.20 mval/kg.

As expected, the values of conductivity were much lower for Manuka samples (as nectar honey). A similar average conductivity value for this type of honey is given by Moniruzzaman et al.^41^, which places it quite close to the upper limit of conductivity typical for nectar honeys.

Compared to honeydew honey, Manuka honey showed less variability, however, significantly fewer samples were analyzed. Great variability in physicochemical parameters of honeydew honeys was previously described for honeydew honeys from the same region^11^ and other sets of samples^12,40,42^. The values of the parameters tested may be influenced by various factors related to the source of honeydew, possible admixtures, as well as climatic factors and those related to the condition of the bee family. Some features (e.g. water content) may also be influenced by the beekeeper’s procedures and honey processing processes^43^.

**Table 2:** Physicochemical parameters of tested honey samples.

Sample	pH	Acidity (mEq/kg)	Conductivity (mS/cm)	Color in Pfund scale (mm Pfund)	Water content (%)	HMF (mg/kg)	Diastase number (DN)
Questionable honey samples							
1	4.26 ± 0.02^a^	21.00 ± 0.71^bc^	0.307 ± 0.000^a^	> 150^j^	17.3 ± 0.2^bc^	23.50 ± 0.01^o^	9.41 ± 0.28^c^
2	4.69 ± 0.01^ef^	22.35 ± 0.11^c^	0.312 ± 0.001^b^	105 ± 1^e^	18.0 ± 0.8^cd^	5.26 ± 0.01^k^	4.62 ± 0.25^a^
Honeydew honey							
3	4.69 ± 0.00^ef^	34.25 ± 3.36^g^	0.920 ± 0.001^g^	104 ± 0^e^	17.9 ± 0.3^cd^	3.24 ± 0.02^e^	14.27 ± 0.46^e^
4	4.75 ± 0.04^f^	39.00 ± 0.00^h^	0.871 ± 0.001^f^	117 ± 0^f^	18.1 ± 0.1^cd^	1.59 ± 0.01^d^	23.44 ± 0.60^h^
5	4.83 ± 0.01^g^	45.25 ± 0.18^i^	0.939 ± 0.001^h^	72 ± 1^a^	18.1 ± 0.3^cd^	3.36 ± 0.01^f^	17.56 ± 0.72^f^
6	4.59 ± 0.00^d^	28.85 ± 0.04^de^	0.957 ± 0.001^i^	95 ± 2^c^	17.7 ± 0.6^cd^	3.14 ± 0.03^d^	18.92 ± 0.68^g^
7	4.83 ± 0.02^g^	31.00 ± 0.00^e^	1.060 ± 0.001^k^	107 ± 0^e^	18.9 ± 0.5^de^	1.27 ± 0.02^c^	23.48 ± 0.79^h^
8	4.46 ± 0.00^c^	24.05 ± 0.04^cd^	0.870 ± 0.001^f^	90 ± 1^b^	18.2 ± 0.1^cd^	4.74 ± 0.01^h^	15.34 ± 0.53^e^
9	5.22 ± 0.03^i^	26.50 ± 0.00^de^	1.155 ± 0.001^m^	107 ± 1^e^	16.0 ± 0.2^ab^	5.05 ± 0.04^j^	12.40 ± 0.30^d^
10	4.98 ± 0.01^h^	24.40 ± 0.00^cd^	0.967 ± 0.001^j^	94 ± 0^c^	19.8 ± 0.2^e^	5.89 ± 0.01^m^	12.54 ± 0.23^d^
11	4.56 ± 0.01^d^	31.30 ± 0.00^e^	0.869 ± 0.001^f^	122 ± 0^g^	17.8 ± 0.6^cd^	0.98 ± 0.01^b^	18.35 ± 0.15^fg^
12	4.88 ± 0.04^g^	30.90 ± 0.07^e^	1.069 ± 0.001^l^	90 ± 0^b^	19.2 ± 0.3^de^	0.88 ± 0.03^a^	14.75 ± 0.10^e^
Mean ± SD*	4.78 ± 0.22^A^	31.55 ± 6.59^B^	0.970 ± 0.100^B^	99.80 ± 14.65^A^	18.17 ± 1.02^A^	3.01 ± 1.81^A^	17.11 ± 4.02^B^
Applicable EU limits (Directive 2014)		< 50	> 0.8		20%	< 40	≥ 8
Manuka honey							
M-1	4.64 ± 0.00^de^	17.05 ± 0.04^a^	0.673 ± 0.001^e^	150 ± 0^i^	16.2 ± 1.0^a^	4.84 ± 0.03^i^	7.39 ± 0.56^b^
M-2	4.37 ± 0.04^b^	18.90 ± 0.07^ab^	0.535 ± 0.001^d^	141 ± 2^h^	17.4 ± 0.6^c^	5.66 ± 0.01^l^	6.72 ± 0.31^b^
M-3	4.39 ± 0.02^b^	17.90 ± 0.00^a^	0.458 ± 0.001^c^	> 150^j^	16.0 ± 0.1^a^	7.49 ± 0.02^n^	5.77 ± 0.10^ab^
M-4	4.33 ± 0.02^b^	20.00 ± 0.07^bc^	0.457 ± 0.001^c^	149 ± 1^h^	15.5 ± 0.3^a^	4.28 ± 0.02^g^	6.47 ± 0.13^b^
Mean ± SD*	4.43 ± 0.14^A^	18.46 ± 1.27^A^	0.531 ± 0.102^A^	147.5 ± 4.36^B^	16.28 ± 0.81^A^	5.57 ± 1.40^A^	6.59 ± 0.67^A^
Applicable EU limits (Directive 2014)		< 50	< 0.8		20%	< 40 < 15^**^	≥ 8 ≥ 3^**^

^a, b,c, d,e, f,g, h,i, j,k, l,m, n,o^Sharing the same letters in the columns are not significantly different (*p* > 0.05).

^A, B^Sharing the same letters for honeydew and Manuka honeys are not significantly different (*p* > 0.05).

*Results are the average ± SD of ten honeydew honey samples (3–12) and four Manuka honey samples.

**Limits permissible for honeys with low natural enzyme content^44^.

### Antioxidant activity and polyphenols content

Antioxidant properties of honeydew honey ranged from 1830.30 to 3656.06 µmol Trolox/kg (Table 2), excluding both questionable samples (1 and 2). The analyzed samples showed high antioxidant potential and were within the range previously determined for samples of Polish honeydew honey: 1443.53-2213.82 µmol Trolox/kg (four samples)^11^ and 1180.77–3701 µmol Trolox/kg (higher number of samples)^7^. The average value of antioxidant properties of Manuka honey was 2802.27 µmol Trolox/kg, which was comparable with the best honeydew honey samples. Similarly, antioxidant properties of New Zealand honeydew honey were comparable to Manuka honey^45^, the observation was also confirmed by Gośliński et al.^46^. On the other hand, Pentoś et al.^47^ observed that the antioxidant potential of Manuka honey was approximately twice as high as that of honeydew honey.

**Table 3:** Phenolic content, antioxidant properties and glucose oxidase activity of tested honeys.

Sample	TPC (mg GAE/kg)	FRAP (µmol Trolox/kg)	Glucose oxidase (GOX) mU/ml
Questionable honey samples			
1	690.7 ± 31.5^ce^	1528.8 ± 25.0^ab^	8.9 ± 0.5^a^
2	405.9 ± 25.6^a^	1271.2 ± 6.9^a^	19.8 ± 0.2^d^
Honeydew honey			
3	632.6 ± 23.5^cd^	2297.0 ± 113.2^cd^	28.4 ± 0.2^h^
4	676.1 ± 17.6^de^	2550 ± 138.4^d^	29.5 ± 1.0^h^
5	964.4 ± 76.6^g^	3656.1 ± 71.3^g^	24.4 ± 0.2^g^
6	919.0 ± 60.3^g^	3043.9 ± 101.3^f^	20.4 ± 0.3^e^
7	597.0 ± 15.2^bc^	2159.1 ± 16.4^c^	31.1 ± 2.2^i^
8	891.2 ± 82.9^g^	2681.8 ± 157.3^def^	18.9 ± 0.5^d^
9	1102.4 ± 79.5^h^	3583.3 ± 155.5^g^	43.5 ± 0.2^j^
10	811.6 ± 41.7^f^	2915.2 ± 137.0^ef^	22.2 ± 0.2^f^
11	583.9 ± 39.3^bd^	1830.3 ± 93.9^b^	18.6 ± 0.2^d^
12	752.44 ± 13.9^e^	2592.4 ± 81.9^cd^	19.2 ± 0.3^de^
Mean ± SD*	793.1 ± 174.6	2730.9 ± 586.4	25.6 ± 7.8
Manuka honey			
M-1	873.9 ± 62.4^g^	2981.8 ± 135.5^f^	17.0 ± 0.2^c^
M-2	839.4 ± 55.1^fg^	2512.1 ± 81.4^cd^	12.9 ± 0.3^b^
M-3	920.5 ± 64.9^g^	2960.6 ± 76.1^f^	12.2 ± 0.7^b^
M-4	790.5 ± 1.7^g^	2754.6 ± 67.0^def^	11.6 ± 0.3^b^
Mean ± SD*	856.1 ± 54.9	2802.3 ± 218.9	13.4 ± 2.5

^a, b,c, d,e, f,g, h,i, j^Sharing the same letters in the columns are not significantly different (*p* > 0.05).

*Results are the average ± SD of ten (3–12) honeydew honey samples and four Manuka honey samples.

Total phenolic content (TPC) of analyzed honey samples ranged from 583.87 to 1102.42 mg GAE/kg, excluding samples 1 and 2 (Table 3). Obtained results are comparable with other authors’ findings. Wilczyńska^48^ obtained comparable results for honeydew honey collected from the same part of Poland (Bieszczady) with the values ranged from 582.4 to 718.4 mg GAE/kg. Similarly, Puścion-Jakubik et al.^49^ found the TPC value of 640–1207 and 455.8 -924.1 mg GAE/kg for coniferous and deciduous honeydew honey, respectively. Similar results were previously described by us^7^, where phenolic content of honeydew honey ranged from 372.97 to 1001.02 mg GAE/kg and by Miłek et al.^33^ with the TPC content ranged from 310 to 646 mg GAE/kg. The lower phenolic content for Polish honeydew honey was determined by Kędzierska-Matysek et al.^50^ with the value around 164.3 mg GAE/kg. Similar results to our study were observed for honeydew honey samples originated from different countries from east Europe. TPC for Spanish honeydew honey was within the range from 550 to 750 mg GAE/kg^51^ whereas for Slovakian honey was from 500 to 899.72 mg GAE/kg^52^, for Serbian forest honeys ranged from 769 to 1052 mg/kg^53^, for Romanian honey ranged from 530.91 to 1960 mg GAE/kg or 1100–2150 mg GAE/kg^54^, for Bulgarian 1184.6-1331.2 mg GAE/kg and for honey from Croatia the TPC content was 945.8–1339.2 mg GAE/kg^55^, while for Czech samples the mean value of TPC was around 300 mg GAE/kg^56^.

Mean TPC value of tested Manuka honey was comparable to honeydew honey. Similarly Gośliński et al.^46^ observed that phenolic content of Manuka honey was quite similar to the phenolic content of honeydew honey; however the established values were tree times higher than those obtained by us. On the other hand, Anand et al.^57^ determined higher phenolic content in Manuka honey (1288 mg/kg GAE) and Pentoś et al.^47^ observed that total phenolic content of honeydew honey was more than twice lower than Manuka honey. The strong correlation was observed between antioxidant potential and phenolic content (*r* = 0.91), which was also previously observed by other authors^9,58^. However, some studies reported a low or negative correlation, which suggests that in some honey types, their antioxidant potential does not depend mostly on phenolic compounds, but other factors, like: amino acids, proteins, organic acids, products of Maillard reaction^58^.

### HPTLC polyphenolic profile

**Fig. 1:**
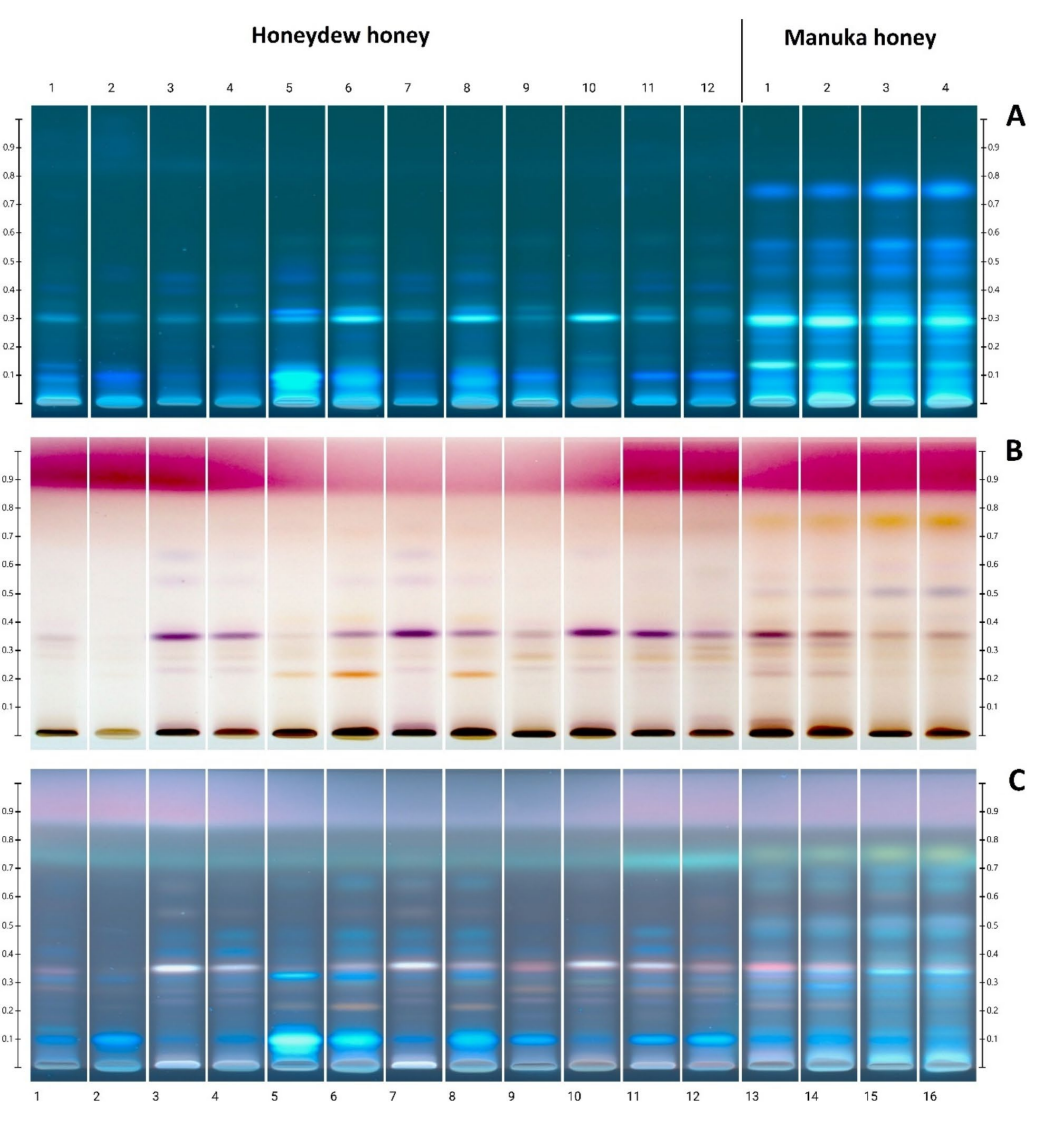
Image of the chromatographic plate: before derivatization in UV 366 nm (**A**), and after derivatization in white light (**B**) and UV 366 nm (**C**). Tracks 1–2: questionable honey samples, 3–12: honeydew honey, 13–16: Manuka honey.

The image of the chromatographic HPTLC plate is shown in Fig. 1: before derivatization in UV 366 nm (A), and after derivatization in white light (B) and UV 366 nm (C). As it was established earlier^11^ for honeydew honeys, a characteristic arrangement of HPTLC bands was identified, using the same separation conditions. It can be concluded that the typical profile for honeydew honey was observed for all samples and was characterized by a characteristic violet in white light and white in UV light after derivatization with a band at Rf = 0.35. This band is much less visible in questionable samples 1, 2 and also 5. However, these conflicting results of the polyphenol profile of samples no. 1 and 2 coincide with very low values of electrical conductivity, which raised questions about the reliability of classification of both samples as honeydew honey. This is consistent with the conclusions drawn from the significance of other parameters for these samples (Tables 2 and 3). Moreover, an additional chromatographic analysis of the sugar profile could be useful, in confirming the honey variety based on the presence or absence of raffinose and melezitose as these sugars are characteristic for honeydew honeys^59^.

Up to now, honeydew honey and Manuka honey have not been directly compared by HPTLC. Manuka honeys show a different profile with additional bands at Rf = 0.74 (yellow) and 0.52 (purple-blue). Thanks to the analysis of images after HPTLC separation, it is possible to conclude that Manuka honeys are distinguished by a specific set of polyphenolic substances because of their botanical origin. Honey of this variety was tested with the same method earlier, thanks to the use of the created database, i.a. specific compounds such as leptosperine, mandelic acid, kojic acid, lepteridine, lumichrome and other polyphenols^60^. In comparative studies with selected varieties of European honeys, the HPTLC profile analysis method supported by PCA showed that Manuka honey shows some similarity to honeydew samples. However, markers distinguishing honeydew honey have been detected as rosmarinic and ellagic acid^61^.

Honeydew honey samples 3, 4, 7, 8 and 9, which later showed the strongest effect on the tested bacteria and bacteriophages, were characterized by the polyphenol profile determined by the HPTLC method, that is most typical for this type of honey, with an intense purple band at Rf = 0.35. On the other hand, the questionable samples 1 and 2 and sample 5, which showed a clearly different HPTLC profile (rich in intensely fluorescent blue bands), performed worse in the tests of antibacterial and antiviral activity. This shows the relationship between the polyphenol composition and the bioactivity of honey. The difference in the polyphenol composition between honeydew and Manuka honey may also translate into their different effects on microorganisms. However, the precise determination of the polyphenol profile and the association of specific compounds with biological effects requires in-depth exploration using coupled with mass spectrometry or bioautographic techniques.

### Glucose oxidase activity and hydrogen peroxide

The glucose oxidase (GOX), an enzyme present in inactive form in honey, is activated after honey dilution. This enzyme is sensitive to light and storage conditions of honey. GOX oxidizes glucose to the gluconic acid and hydrogen peroxide. The GOX activity in tested honeydew honey ranged from 18.64 to 43.50 mU/ml, with the highest values obtained for samples no 3,4,7 and 9 and the lowest observed for sample no 1 (8.87 mU/ml). The values obtained for all Manuka honey were significantly lower. Similar values of GOX activity were described by Bucekova et al.^52^ for honeydew honey (from 21 to 50 mU/ml), whereas lower was found for Caledonian honey (from 0.1 to 13.34 mU/ml)^13^. The very low activity of GOX in Manuka honey was also reported previously by Bucekova et al.^52^. The authors suggested that methylglyoxal present in Manuka honey may structurally modify the GOX enzyme.

**Fig. 2:**
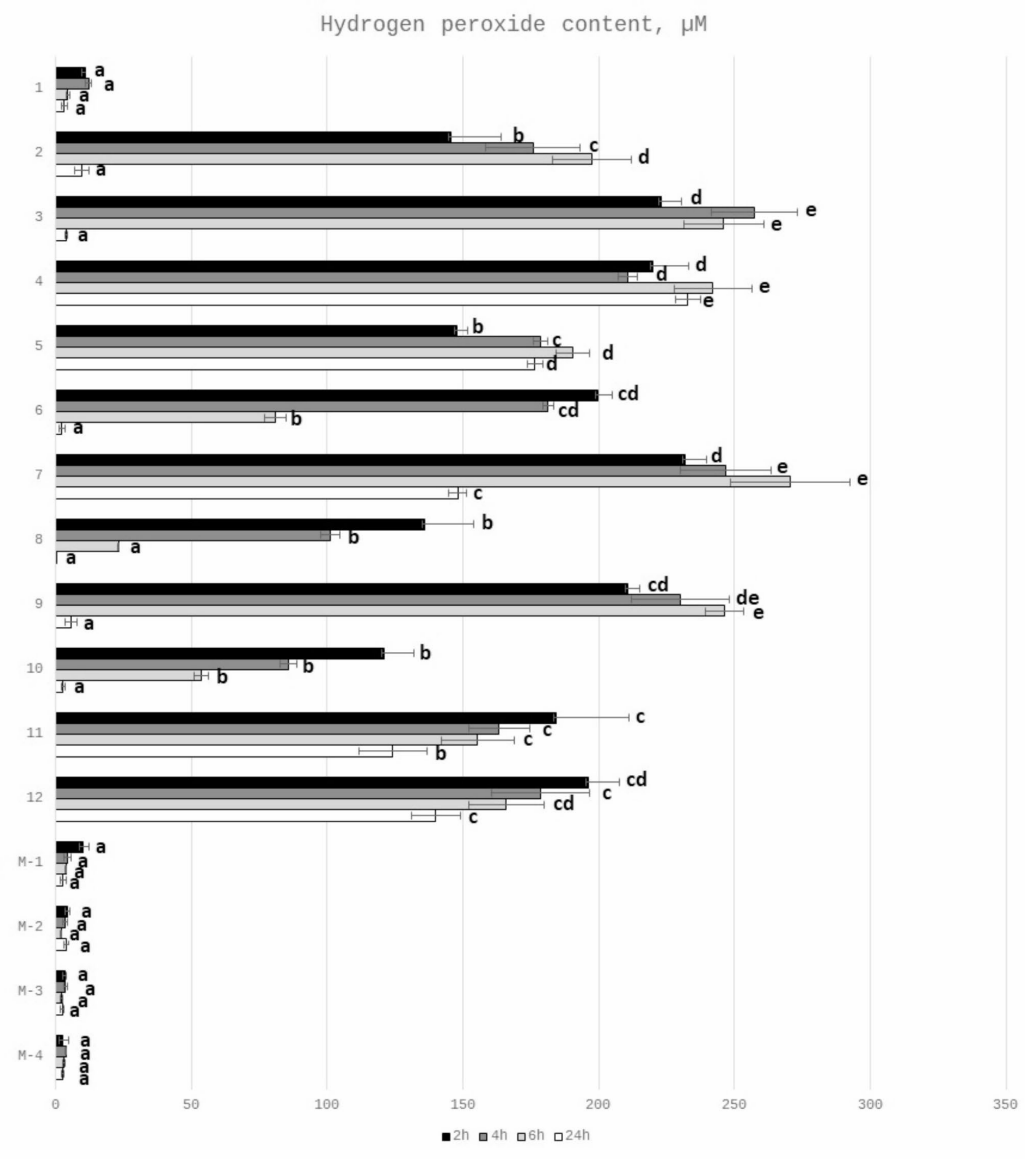
Hydrogen peroxide content in 25% (w/v) honey solutions regarding different times of their incubation at room temperature. a, b, c, d, e means sharing the same letter (within one time) are not significantly different from control (*p* > 0.05).

The hydrogen peroxide (H_2_O_2_) is considered as a one of the important factors influencing the antibacterial activity of honey, which was proved by decreased antibacterial properties after catalase treatment of honey samples^25,62–69^. Hydrogen peroxide is obtained during activity of the enzyme present in honey – glucose oxidase. This enzyme is inactive in full ripened honey, while after honey dilution, the glucose oxidase converts glucose present in honey into gluconic acid and hydrogen peroxide. The content of hydrogen peroxide depends on the type of honey, honey age, storage conditions, honey dilution rate and also time since the honey dilution was prepared^26^. Therefore, the concentration of hydrogen peroxide was tested after different incubation times (after 2,4,6 and 24 h). The content of H_2_O_2_ (determined in 25% honey solutions) of the tested honey samples is presented in Fig. 2. It can be observed that there are differences between samples in the time of the incubation needed for the maximum production of H_2_O_2_. For honeydew honey samples significant correlation between GOX activity and H_2_O_2_ content was observed (*r* = 0.679, *p* < 0.05). (Table 3). For most honey samples the highest content of hydrogen peroxide was generated after 4–6 h of incubation time, which was previously reported by Lehmann et al.^26^ and Bucekova et al.^13^. They observed that kinetics of generation and degradation of hydrogen peroxide differs among honey samples with the highest concentration obtained after 3–6 h of honey dilution. The content of hydrogen peroxide after 24 h of incubation in most samples decreased dramatically. Only for samples no 3,4,5,7 the content of H_2_O_2_ was still very high after this incubation time. As it was expected the content of hydrogen peroxide in Manuka honey was at a very low level. It is known that production of hydrogen peroxide is relatively low in Manuka honey and, additionally, can be neutralized by a catalase^14^. Antibacterial actions of honeydew and Manuka honey involve two different mechanisms: hydrogen peroxide dependent (in honeydew honey) and non-peroxide antimicrobial activity (in Manuka honey)^70,71^. It is closely associated with the floral sources, mainly from *Leptospermum* species and MGO content^72,73^ and was fully supported by our results that the determined hydrogen peroxide content in analyzed Manuka samples was much lower than that in honeydew honey (Fig. 2). The concentration of H_2_O_2_ (determined in 50% honey solutions) in two honeydew honey samples from Slovakia was at the level of 306.9 and 495.8 µM, while for Manuka honey was 78.9 µM, which is 4 to 6 times lower than in honeydew honey. Hydrogen peroxide content of honeydew honey determined by Bucekova et al.^67^ in 50% diluted honey was around 1200 µM, which was higher than obtained by us. On the other hand, in our previous paper the H_2_O_2_ content of some honeydew honey samples collected from Podkarpacie was around 165 µM after 4 h of incubation^25^. The differences in the results obtained by other authors can be explained by the fact that hydrogen peroxide production is not linear with honey dilutions and appear in a narrow range, usually between 30 and 50% v/v^74,75^. Authors determined the H_2_O_2_ content at different concentrations of honey and after different incubation time, which may have impact on its content^26^. In dark honeys the peak of hydrogen peroxide production occurred at higher dilutions of honey (between 6.25 and 12.5%)^68^. Moreover, it is possible that other honey constituents of botanical origin (known or unknown) may alter H_2_O_2_ levels and their interactions can result in augmentation of honey antibacterial activity^52^. Brudzynski^69^ discussed the possible relationship between the ability to form colloidal structures of honey macromolecules and the hydrogen peroxide production.

### Antibacterial properties

#### MIC and MBC determination

The study with four model bacteria allows us to compare both MIC (Minimal Inhibitory Concentration) and MBC (Minimal Bactericidal Concentration) values for all tested honeys (Table 4). The highest antibacterial properties were observed against Gram-positive bacteria with the mean values of MIC 20.5% and 21.00%, against *S. aureus* and *B. cereus*, respectively. Moreover the lowest values were observed for questionable samples 1 and 2. The most resistant bacteria was *E. coli* with the mean value of MIC at the level 22.50%, which was also observed for Zantaz honey^76^. The MBC was generally higher, with the highest value observed for *B. cereus*.

The highest antibacterial potential was observed for Manuka honey (MIC value against *S. aureus* – 8–15%, mean = 10.25%), however it was also less effective against *E.coli.* Antibacterial potential of Manuka honey depended on the content of MGO. The highest antibacterial potential was observed for honey with the highest MGO content (M-3, M-4; >400 and > 550 respectively), while the lowest was observed for Manuka honey with lower content of MGO (M-1, M-2; 100 and 250 MGO, respectively).

**Table 4:** Minimal inhibitory concentration (MIC 90) and minimal bactericidal concentration (MBC) for tested honeydew and Manuka honeys.

Honey	*S. aureus* ATCC 25923		*B. cereus* PCM 482		*E. coli* NCTC 1893		*S. enterica* NCTC 12634	
	MIC	MBC	MIC	MBC	MIC	MBC	MIC	MBC
Questionable honey samples								
1	30	30	30	35	30	> 50	30	50
2	35	35	35	45	35	40	35	> 50
Honeydew honey								
3	20	20	20	25	25	25	20	20
4	15	15	15	20	20	20	20	20
5	25	25	25	35	30	30	25	25
6	20	20	20	30	25	25	25	25
7	20	20	20	25	20	20	20	30
8	25	25	25	30	25	30	20	30
9	20	20	25	30	25	30	20	20
10	20	20	20	25	20	30	25	25
11	20	20	20	25	15	15	20	20
12	20	20	20	25	20	20	20	20
Mean ± SD*	20.5 ± 2.84	20.5 ± 2.84	21.00 ± 3.16	27.00 ± 4.22	22.50 ± 4.25	24.5 ± 5.5	21.5 ± 2.42	23.5 ± 4.12
Manuka honey								
M-1	15	15	15	20	20	25	20	20
M-2	10	15	10	15	15	20	15	15
M-3	8	10	8	10	15	20	15	15
M-4	8	10	8	10	15	15	15	15
Mean ± SD*	10.25 ± 3.30	12.5 ± 2.87	10.25 ± 3.30	13.75 ± 4.79	16.25 ± 2.5	20 ± 4.08	16.25 ± 2.5	18.75 ± 4.79

*Results are the average ± SD of twelve honeydew honey samples and four Manuka honey samples.

The antibacterial potential of Manuka honey was stronger against Gram-positive bacteria, which was also described earlier by Johnston et al.^77^. These authors also observed that antibacterial potency of Manuka honey is correlated with the methylglyoxal and total phenols content, which was also observed by us. The lower range of MIC values - from 2 to 12.5% of 23 honeydew honey samples against *S. aureus* was found by Bucekova et al.^52^. They also observed that antibacterial potential of honey was stronger against Gram-positive bacteria (*S. aureus*) than Gram-negative bacteria (*Pseudomonas aeruginosa*). According to these authors, the values of MIC and MBC were comparable. Moreover, Manuka exhibited comparable or less effective antibacterial activity as honeydew honey samples. Stronger antibacterial potential against Gram-positive, than Gram-negative bacteria was observed by many authors, as well as for honeydew honey^40^ and also for blossom honey^15,40,78,79^. In turn, MIC values for pine honeydew honeys from Greece against *S. aureus* were lower than obtained by us for Polish honeydew honey, ranging from 3.125 to 12.5% (v/v) whereas for Manuka honey used for comparison, the MIC was 3.125^40^. The reason for the differences in obtained results may be the fact that the authors used Manuka honey with the strongest antimicrobial properties available on the market, with 1122 mg/kg of MGO content. The MIC values of Spanish honeydew honey ranged from 10 to 20%^80^, while for Czech samples the MIC values against *S. aureus* ranged from 5.5 to 22.22%, while MIC for Manuka honey was within this range and reached 11.11% value^56^. The cited studies justify the correctness of our results and also confirm that Podkarpackie honeydew honey exhibits mostly comparable properties to honeys from other countries.

#### Bacterial growth inhibition

**Fig. 3:**
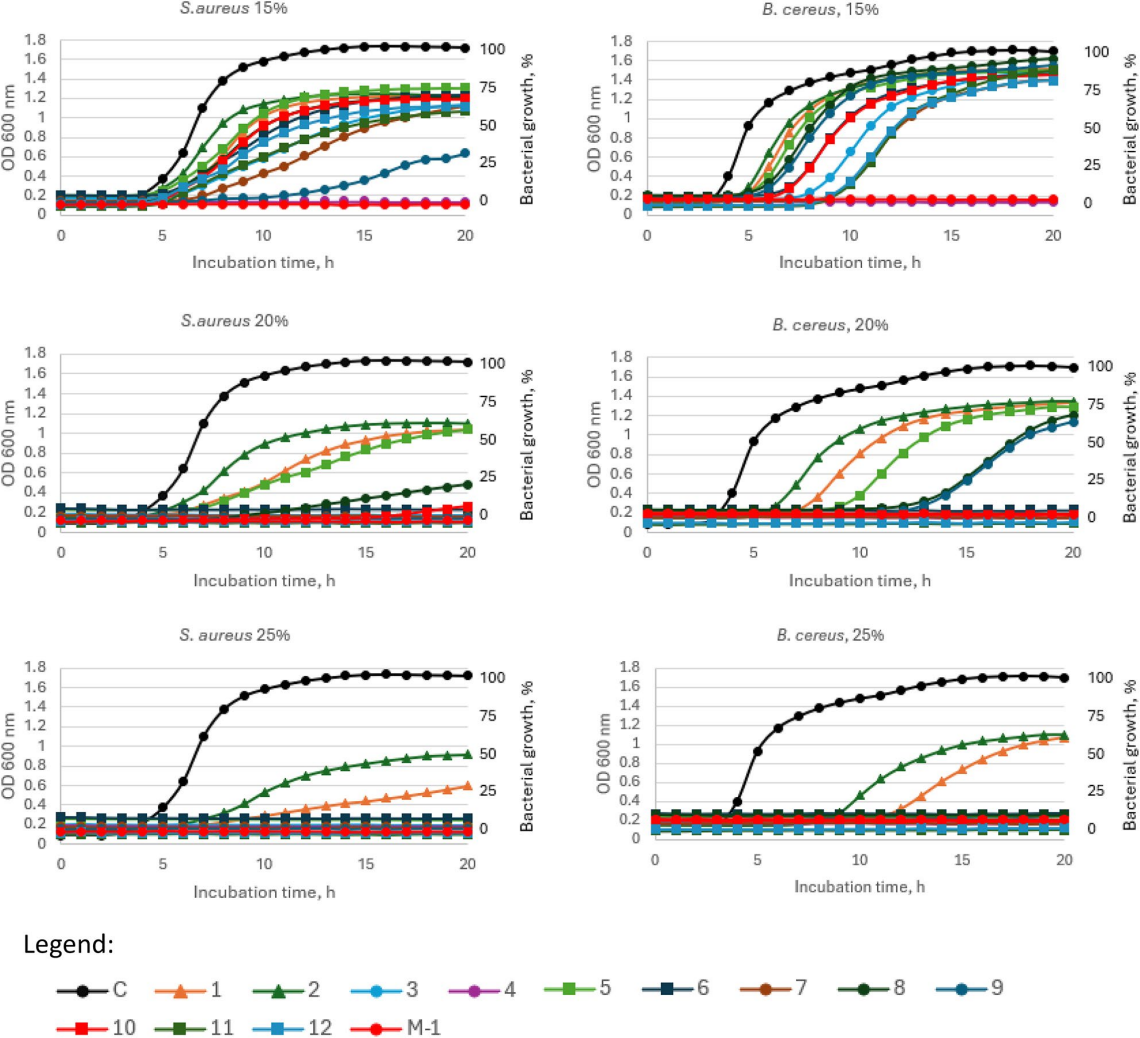
Growth curve of *S. aureus* and *B. cereus* during incubation with 15%, 20% and 25% (w/v) honey concentrations. C—control (*S. aureus* or *B. cereus)*, 1–2—questionable honey samples, 3–12—honeydew honey, M-1—Manuka honey 100 MGO. Legend: Control - Black filled circle, 1 - orange filled triangle, 2 - dark green filled triangle, 3 - blue filed circle, 4 - purple filled circle, 5 - light green filled square, 6 - dark blue filled square, 7 - brown filled circle, 8 - dark green filled circle, 9 - dark blue filled circle, 10 - red filled square, 11 - dark green filled square, 12 - blue filled square, M-1 - red filled circle.

The growth curves of bacteria in the presence of different concentrations of honey (15, 20, 25% w/v) were presented in Fig. 3 (Gram-positive bacteria) and 4 (Gram-negative bacteria). All tested honey samples exhibited antibacterial potential in the dose dependent manner, however some differences between honey sample and bacterial strain used were observed. Manuka honey was effective against Gram-positive bacteria at a concentration of 15%, while only one sample of honeydew honey (no 4) exhibited similar antibacterial properties. Increasing the honey concentration to 20% causes that samples no 3, 4, 7, 9, 11 and 12 strongly inhibited bacterial growth, while at 25% concentration only three samples (1, 2 and 5) were not fully effective (Fig. 3).

Total inhibition of Gram-negative bacteria growth was observed at the 20% honey concentration for Manuka honey and also for honeydew honey samples no 4, 7, 10 and 12 (for *E. coli*) and no 3, 4, 7, 9 11 and 12 (for *S. enterica*). An increase of honey concentration to 25% caused that only samples 1 and 2 did not completely inhibit the growth of *Salmonella* and samples 1, 2 and 5 in the case of *E. coli.*

The highest antibacterial potential was observed for samples no 3,4, and 7,8. Generally, at the 25% honey concentration most of the tested honey samples inhibited the bacterial growth > 90% of all tested bacteria and the strongest antibacterial properties were observed for *S. aureus*. The most deviant (with the lowest bacterial inhibition growth) were samples no 1, 2 and 5 which have been earlier pointed as poor quality. At a low concentration of honey, the mean inhibition of *B. cereus* was 19%, while at 20% solution of honey, seven samples inhibited bacterial growth > 90%. The most resistant bacteria was *E. coli*, with the average inhibition around 27 and 55% (in the presence of 15 and 20% honeydew honey, respectively).

Manuka honey at 15% concentration inhibited Gram-positive bacteria at 90–93%. The lowest inhibition was observed against Gram-negative bacteria – 27.5 and 57.3%, for *E. coli* and *S. enterica*, respectively.

**Fig. 4:**
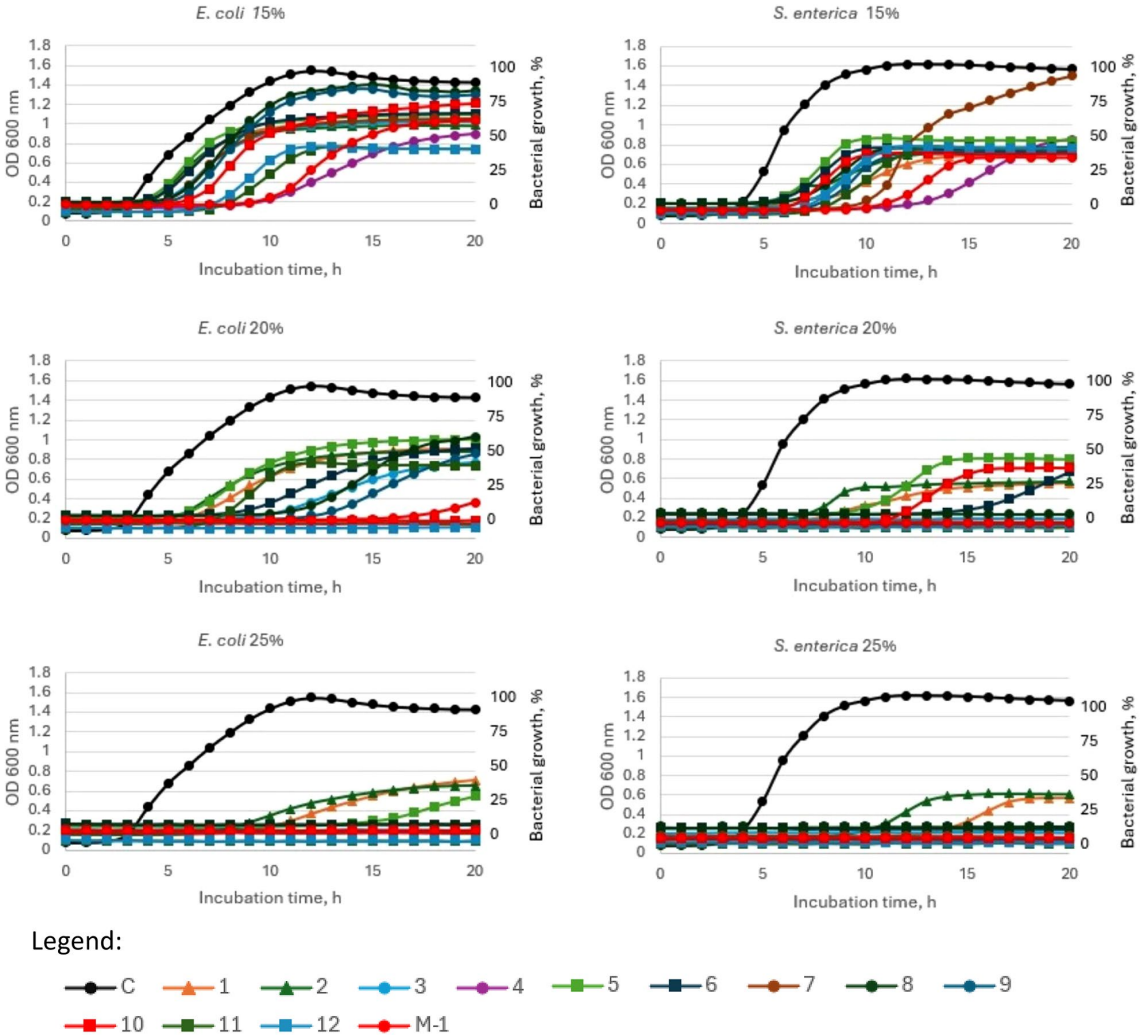
Growth curve of *E. coli* and *S*. enterica during incubation with 15%, 20% and 25% (w/v) honey concentrations. C—control (*E.coli or S. enterica*), 1–2—questionable honey samples, 3–12—honeydew honey; M-1—Manuka honey 100 MGO. Legend: Control - Black filled circle, 1 - orange filled triangle, 2 - dark green filled triangle, 3 - blue filed circle, 4 - purple filled circle, 5 - light green filled square, 6 - dark blue filled square, 7 - brown filled circle, 8 - dark green filled circle, 9 - dark blue filled circle, 10 - red filled square, 11 - dark green filled square, 12 - blue filled square, M-1 - red filled circle.

Manuka honey exhibited strong antibacterial potential, however sample 4 of honeydew honey was as active as Manuka honey even at the lowest concentration of honey − 15% (*p* > 0.05). In turn, at 25% concentration all honeydew honey samples showed similar antibacterial activity to Manuka honey whereas samples 1 and 2 (negative controls) showed a significantly different effect compared to Manuka (*p* < 0.01 and *p* < 0.001, respectively). It can be observed that honey induced the lag phase of cell growth, which was also observed previously for Manuka honey^81^. The antibacterial potential of honey was correlated with FRAP (*r* = 0.57), indicating the contribution of honey’s antioxidant compounds to its antibacterial activity. Similar correlation was observed for Australian honeys^82^ (Fig. 4).

#### Antiviral potential of honey

Bacteriophages phi6 and MS2 (RNA viruses) are attractive surrogates for the determination of antiviral potential and viral inactivation of honey, because most medically important human viruses are RNA viruses^17^. Bacteriophages are readily cultivatable, with rapid enumeration (24 h or less), and inexpensive analysis. Moreover, there are not pathogenic to humans and therefore the analysis can be done in high numbers without the need for additional safety measures^20–22^.

**Fig. 5:**
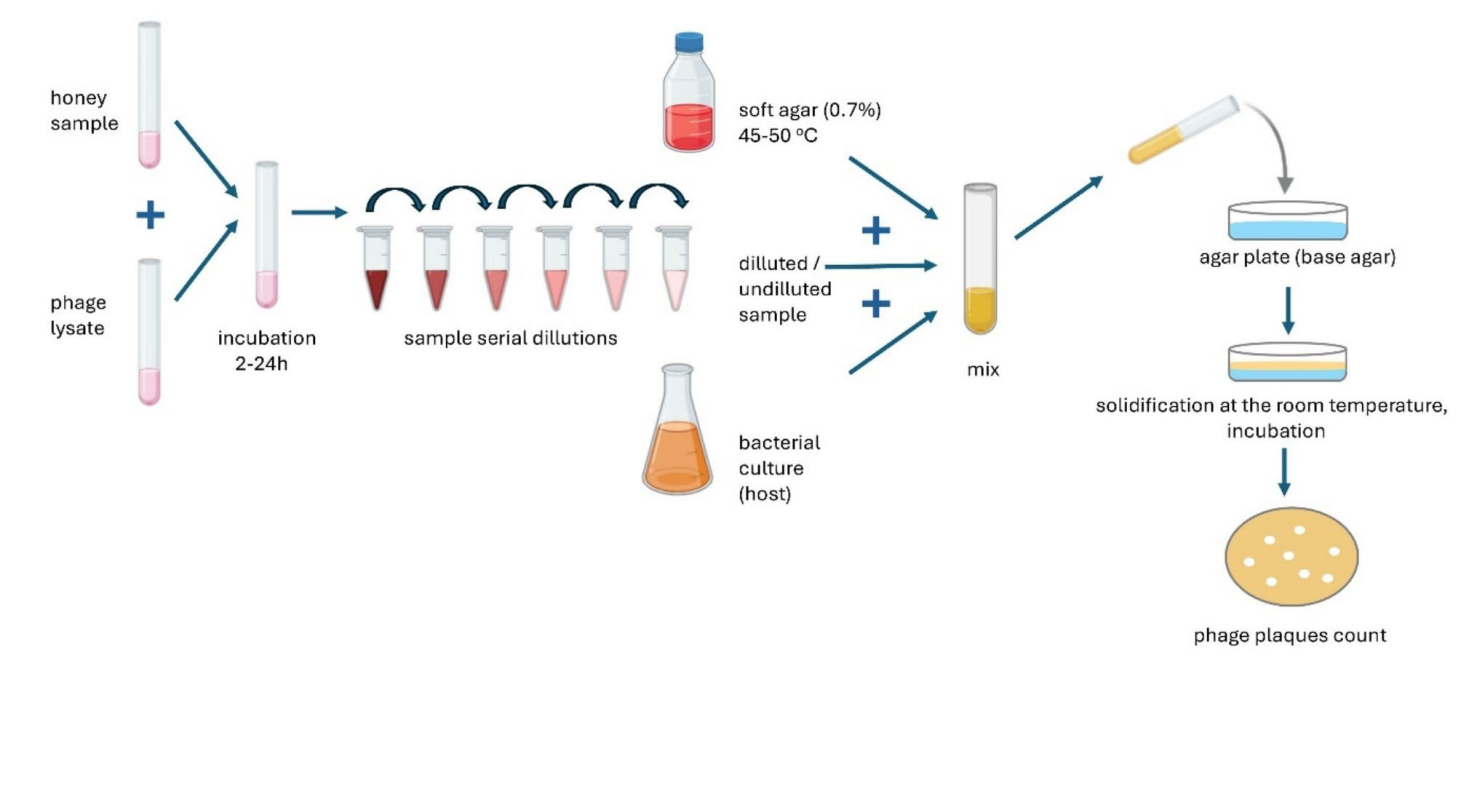
Double-layer phage assay technique for enumeration of virus surrogates.

**Fig. 6:**
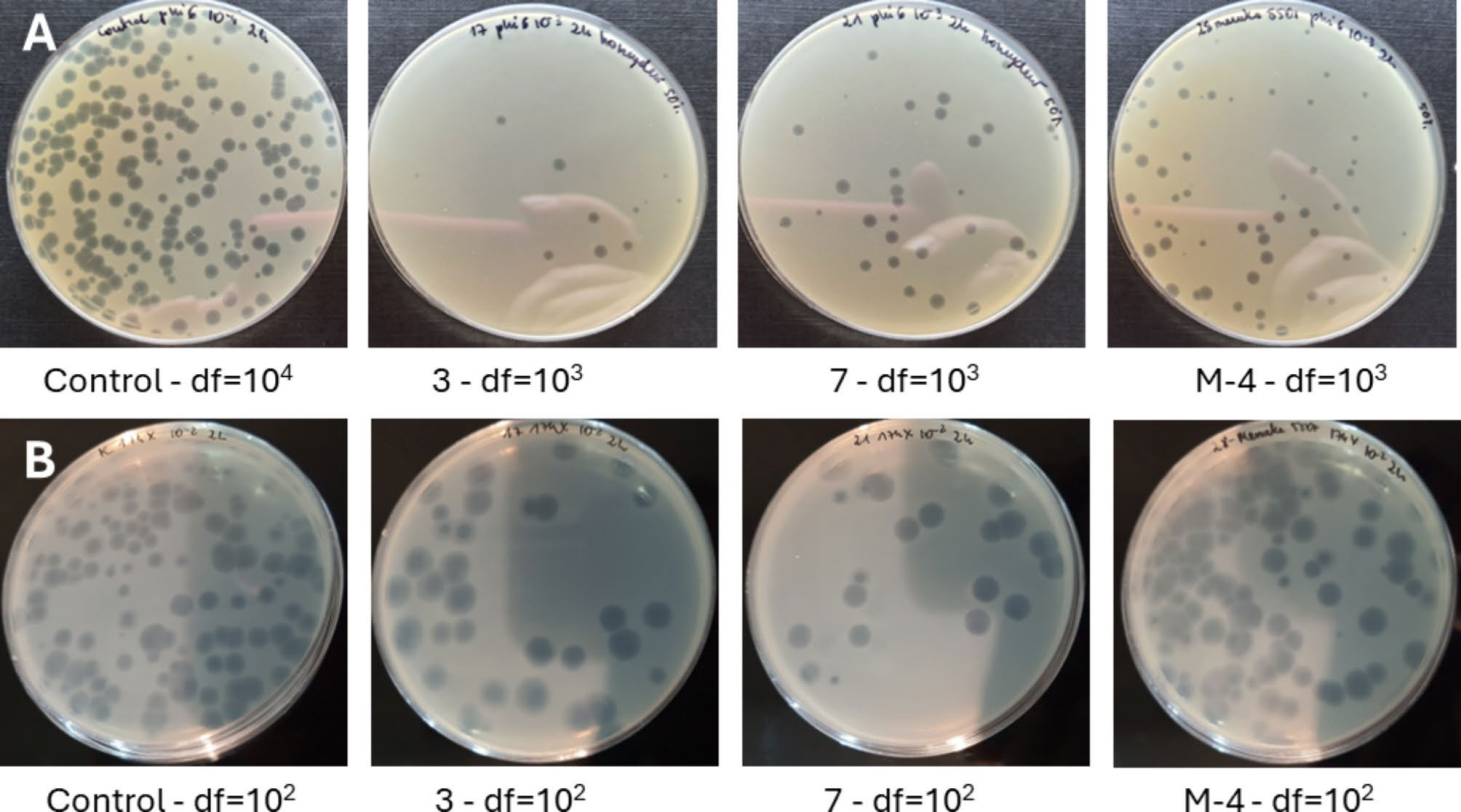
Viral particles reduction after 2 h of incubation with 50% honey samples. (**A**) Bacteriophage phi6; (**B**) bacteriophage phiX174. *Control* samples without honey, *3*,*7* honeydew honey, *M4* Manuka honey, *df* dilution factor.

The antiviral potential of honey was determined by using double-layer phage assay technique (Fig. 5), and results are presented in Table 5. The strong reduction of viable viral particles of phi6 bacteriophage was observed for Manuka honey samples with the highest MGO content (M-3 and M-4). Some of honeydew honey samples (no 3,4,7 and 9) were characterized by the very strong antiviral potential against tested bacteriophages, similar to Manuka honey (*p* > 0.05). Specifically, inhibition of bacteriophage phi6 was very promising as it was used as a “surrogate” organism for determination of antiviral potential against SARS-CoV-2. In this case, the antiviral mechanism of action is often associated with disruption of the viral membrane of the lipid envelope^83^. The most resistant bacteriophage was phiX174, which is a DNA virus. The reduction of viable viral particles was within the range from 1.37 to 2.71 log_10_ PFU/ml. However, the strongest potential was observed for the samples no 3,4,7 and 9 again, which was comparable with the results obtained for phi6 bacteriophage. Manuka honey inhibited phiX174 bacteriophage at the level of around 2 logs, which was at the similar or slightly lower level in comparison with the strongest honeydew honey samples. Very promising results were obtained for RNA virus, MS2. It was observed that most of the honeydew honey samples expressed higher antiviral potential, than Manuka honey. Manuka honey inhibited this RNA virus at a similar level as the samples no 1 and 2 with the lowest bioactivity and questionable quality. The detailed antiviral effect of honey against MS2 virus and the mechanisms of action will be analyzed in the future. The visualization of the reduction of phage plaque formation (phi6 and phi174X) after incubation with honey is shown in Fig. 6. To better understand the strong antiviral potential of the tested honey, the reduction of viral particles presented in logarithmic units can be converted to percentage values. A reduction of 1 log of viral particles corresponds to a reduction of 90% of viral particles (when 100% is the number of bacteriophage particles in the control sample - without honey addition), 2 logs − 99% of viral particles reduction, 3 logs – 99.9% and 4 logs – 99.99% of viral particles reduction, etc.

A little is known about antiviral potential of honey, and according to our knowledge the antiviral potential of honeydew honey with bacteriophages usage as viral surrogates was analyzed for the first time. However, in our previous studies, the antiviral potential of rape honey enriched with different plant extracts (blackberry and raspberry)^27^ and *Aronia melanocarpa*^84^ against phi6 was also observed. Antiviral potential of MGO (present in Manuka honey) against T4 bacteriophage was also observed by Van Buskirk et al.^85^, which suggests that Manuka honey can also inhibit T4 bacteriophage. The strong antiviral potential of honeydew honey samples is very promising due the growing demand for new drugs with antiviral properties and a high risk of new viral epidemics in the coming years.

**Table 5:** Antiviral potential of honey against four bacteriophages after 24 h of incubation.

	Reduction log_10_ PFU/ml			
	Phi6	MS2	T7	PhiX174
Questionable honey samples				
1	1.50 ± 0.20^ab^	0.64 ± 0.18^a^	1.78 ± 0.11^ab^	1.43 ± 0.21^a^
2	0.62 ± 0.02^a^	0.75 ± 0.06^a^	1.76 ± 0.01^ab^	1.37 ± 0.11^a^
Honeydew honey				
3	4.95 ± 0.32^d^	4.33 ± 0.51^c^	5.7 ± 0.29^d^	2.49 ± 0.21^ab^
4	≥ 6.59 ± 0.45^e^	4.55 ± 0.05^c^	≥ 9.38 ± 0.0^e^	2.71 ± 0.16^b^
5	2.39 ± 0.12^bc^	2.72 ± 0.13^b^	1.43 ± 0.8^a^	1.55 ± 0.62^ab^
6	3.39 ± 0.03^cd^	4.14 ± 0.49^c^	3.53 ± 0.6^bc^	2.13 ± 0.37^ab^
7	5.52 ± 0.07^d^	≥ 5.87 ± 0.00^d^	≥ 9.38 ± 0.0^e^	1.96 ± 0.56^ab^
8	3.31 ± 0.04^c^	4.09 ± 0.11^c^	5.02 ± 0.58^cd^	2.17 ± 0.28^ab^
9	5.10 ± 0.01^d^	≥ 5.87 ± 0.00^d^	5.45 ± 0.36^cd^	2.34 ± 0.28^ab^
10	3.86 ± 0.16^cd^	4.78 ± 0.55^c^	1.34 ± 0.63^a^	2.04 ± 0.32^ab^
11	3.70 ± 0.15^c^	4.69 ± 0.62^c^	4.74 ± 0.08^cd^	2.08 ± 0.13^ab^
12	2.46 ± 0.61^bc^	2.40 ± 0.20^b^	4.20 ± 0.29^cd^	1.81 ± 0.14^ab^
Manuka honey				
M-1	4.51 ± 0.13^d^	0.50 ± 0.33^a^	1.96 ± 0.25^a^	2.09 ± 0.27^ab^
M-2	4.80 ± 0.25^d^	0.61 ± 0.30^a^	2.9 ± 0.36^a^	2.02 ± 0.31^ab^
M-3	≥ 6.59 ± 0.00^e^	0.92 ± 0.40^a^	3.00 ± 0.23^a^	2.15 ± 0.27^ab^
M-4	≥ 6.59 ± 0.00^e^	0.89 ± 0.35^a^	2.98 ± 0.22^a^	2.21 ± 0.46^ab^

^a, b, c, d, e^Sharing the same letters in the columns are not significantly different (*p* > 0.05).

The results above showed that some of the tested honey samples strongly inhibited the formation of viral plaques after 24 h of incubation with honey. Consequently, for honey samples with the strongest antiviral potential the influence of the time of bacteriophages incubation with honey on the survival of phages was further investigated.

**Fig. 7:**
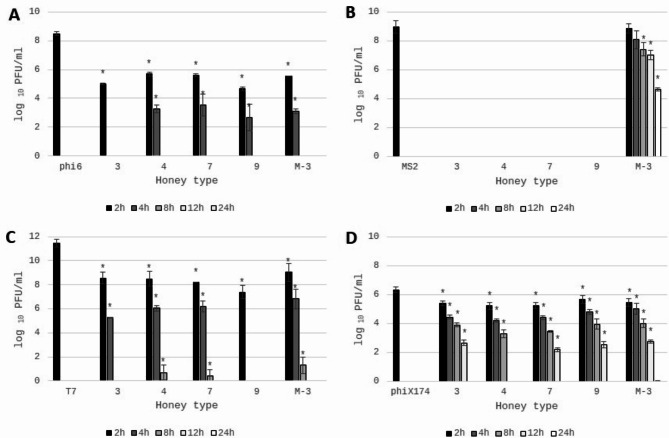
Antiviral potential of honey samples against different bacteriophages: (**A**)—phi6; (**B**)—MS2; (**C**)—T7; and (**D**)—phiX174. 3,4,7,9—honeydew honey, M-3—Manuka honey. Plaque-forming units (PFU) were calculated after 2,4,8,12,24 h of honey extract (50%) incubation with bacteriophages. phi6, MS2, T7, phiX174 bars—positive controls (PFU of viral particles in STM buffer without addition of honey). *Sharing asterisk symbol are significantly different from control (*p* < 0.05) (t test); no columns on the graph mean complete virus inhibition by the tested extracts.

The antiviral potential of honeydew and Manuka honey after different times of incubation was shown in Fig. 7. It can be observed that honeydew honey samples inhibit the tested viruses in various ways. The strongest antiviral properties were observed for MS2 virus (Fig. 7B). For all honeydew samples the virus particles were totally inhibited after 2 h of contact with 50% of honey. In opposite, the antiviral properties of Manuka honey were much lower than honeydew honey (*p* < 0.001). Very strong inhibition of viral particles was observed also for phi6 bacteriophage (Fig. 7A). After 2 h of honey contact the reduction of viral particles was comparable, with the value above 3 logarithmic units (compared to the control) for all tested honeydew samples, while after another 2 h reduction grew to 5 logs for 3 of 4 honey samples, while one sample totally inhibited survival of viral particles. The action of honeydew honey was comparable to Manuka honey.

Strong inhibition of viral particles was also observed for DNA virus – T7, with the reduction level of 2.91–4.07 logs after 2 h, increasing to 5–6 logs after 4 h of contact. Contact for 8 h with honey almost totally inhibited the vitality of T7 virus for all analyzed honey samples (Fig. 7C). The highest resistance of used bacteriophages for honey samples was observed for phiX174 (Fig. 7D). After 2 h of viral contact with honeydew honey the number of viral particles was reduced only 0.66 to 1 log. However, prolonged incubation positively influenced antiviral potential. After 4 h, 6 h, 12 h of incubation, honeydew honey samples inhibited viral survival of around 1.5-2 logs, 2.4-3.0 logs, 4 logs, respectively. Only one honeydew sample (no 4) totally inhibited the phiX174 particles survival after 12 h of incubation. Similarly, very weak antiviral potential against virus phiX174 was observed for Manuka honey. To successful reduction of virus phiX174, long contact time and high content of honey must be used.

In summary, the honeydew honey expressed very strong antiviral potential, but it was demonstrated that honey inhibited the tested viruses in various ways and expressed similar or even higher antiviral potential against viral surrogates chosen for this experiment than well-known Manuka honey. Observed differences result probably from various chemical compositions of tested honey samples and it is very interesting to analyze the exact chemicals responsible for antiviral potential of honeydew and Manuka honey. The highest antiviral potential was observed for honey no 3,4,7 and 9 with the highest glucose oxidase activity and the highest hydrogen peroxide content, which may suppose that these compounds significantly affect the antiviral potential of honeydew honey. However, the statistically significant correlation was observed only for GOX activity (*r* = 0.72, 0.64, 0.61 and 0.57 for MS2, phi6, T7 and phiX174, respectively), but not for H_2_O_2_ content. Similarly, Yamaguchi et al.^86^ described the virucidal effect of glucose oxidase on human immunodeficiency virus type 1 (HIV-1). However, virucidal activity of hydrogen peroxide was described previously against both the enveloped Herpes Simplex virus, and the non-enveloped polio virus^87^ and lack of statistically significant correlation can be caused by many of the factors mentioned above, that can affect hydrogen peroxide levels in honey^68,69^. Statistically important correlation, with high correlation coefficients, was observed between antibacterial potential of honey and antiviral potential against RNA viruses (r = 0.72 and 0.84 for phi6 and MS2 viruses, respectively), which may suggests that the same compounds present in honey are responsible for its antibacterial and antiviral properties. Statistically significant correlations were also observed between bacteriophages used (*r* = 0.58–0.69), which allows us to suppose that honey with strong antiviral properties against one bacteriophage will also have strong antiviral properties against other bacteriophages. However, research confirming these assumptions must be performed. On the other hand, phenolics and flavonoids, which are present in honey exhibit antiviral potential also and may play an important role in determining the virucidal potential of honey^88^, but no correlation was observed. High content of microelements in honeydew honey can be also an important factor influencing its antiviral activity. It has been known that some metals like Mg, Cu, Mn play an important role in the survival and pathogenesis of a large group of viruses^88^. Metal excess can cause inhibition of virus production and this aspect should be analyzed in the future.

## Conclusion

For the first time the strong antiviral potential of coniferous honeydew honey was determined using four bacteriophages: two RNA bacteriophages (phi6 and MS2) and two DNA bacteriophages (T7 and phix174). It was demonstrated that honeydew honey may be important to fight against viral infections. Moreover, the antibacterial properties of honeydew honey best samples were comparable to Manuka honey. The observed similarities between coniferous honeydew honey and Manuka honey are highly significant for economic reasons, as coniferous honeydew honey is much cheaper and more widely available, being harvested in certain parts of the UE. Our findings provide new information and align with the search trend for alternative strategies to combat viral diseases which are becoming increasingly important. Furthermore, the study demonstrates, that the implementation of phages as “viral surrogates” might be a very useful and cost-effective way for initial screening of honey antiviral potential. However, the results of the most promising honey samples should be confirmed against human viruses. Moreover, *in vitro* studies provide preliminary data that need to be verified in clinical trials to establish effective doses *in vivo*.

## Data Availability

Data is provided within the manuscript.
